# Supplementary figures and images for: MprF-mediated immune evasion is necessary for Lactiplantibacillus plantarum resilience in the Drosophila gut during inflammation
Source: PLoS Pathog. 2024 Aug 19;20(8):e1012462. doi: 10.1371/journal.ppat.1012462 (PMC11361745; doi:10.1371/journal.ppat.1012462)

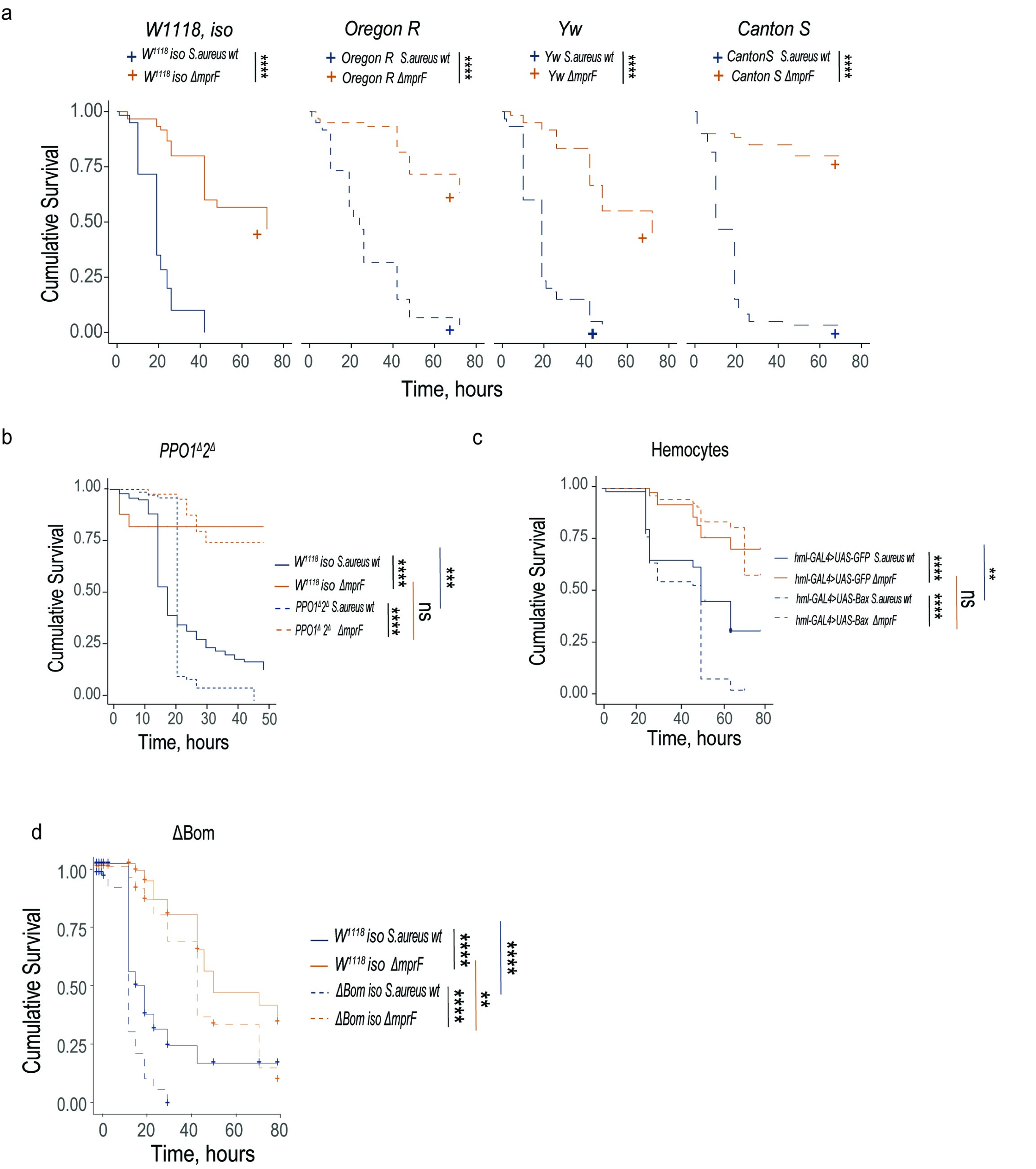

Supplement: S1 Fig — (a) Survival rates of Drosophila wild-type strains infected with wild-type S. aureus or S. aureus ΔmprF mutant. (b, c, d) Survival rates of melanisation-deficient mutant (PPO1Δ2Δ) (b), hemocytes-depleted flies (c), and Bomanins-deficient flies (d) infected with wild-type S. aureus or S. aureus ΔmprF mutant. Each survival graph shows cumulative results of three independent experiments. (TIF) [file ppat.1012462.s001.tif]

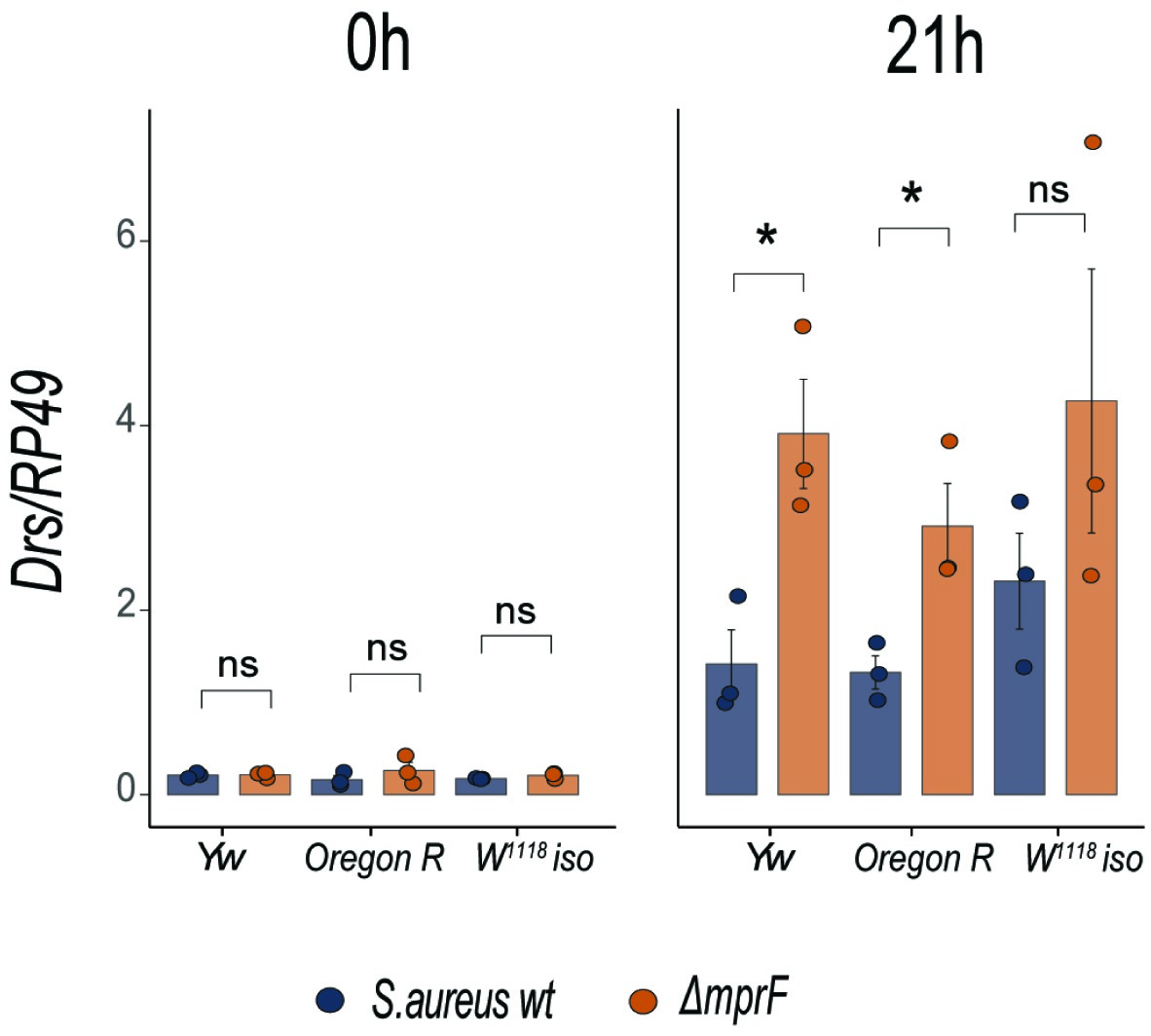

Supplement: S2 Fig — Drosomycin (Drs) gene expression in flies of various genetic backgrounds infected with S. aureus wild-type and S. aureus ΔmprF mutant. Each sample contains 5 animals, n = 3 per each treatment group. (TIF) [file ppat.1012462.s002.tif]

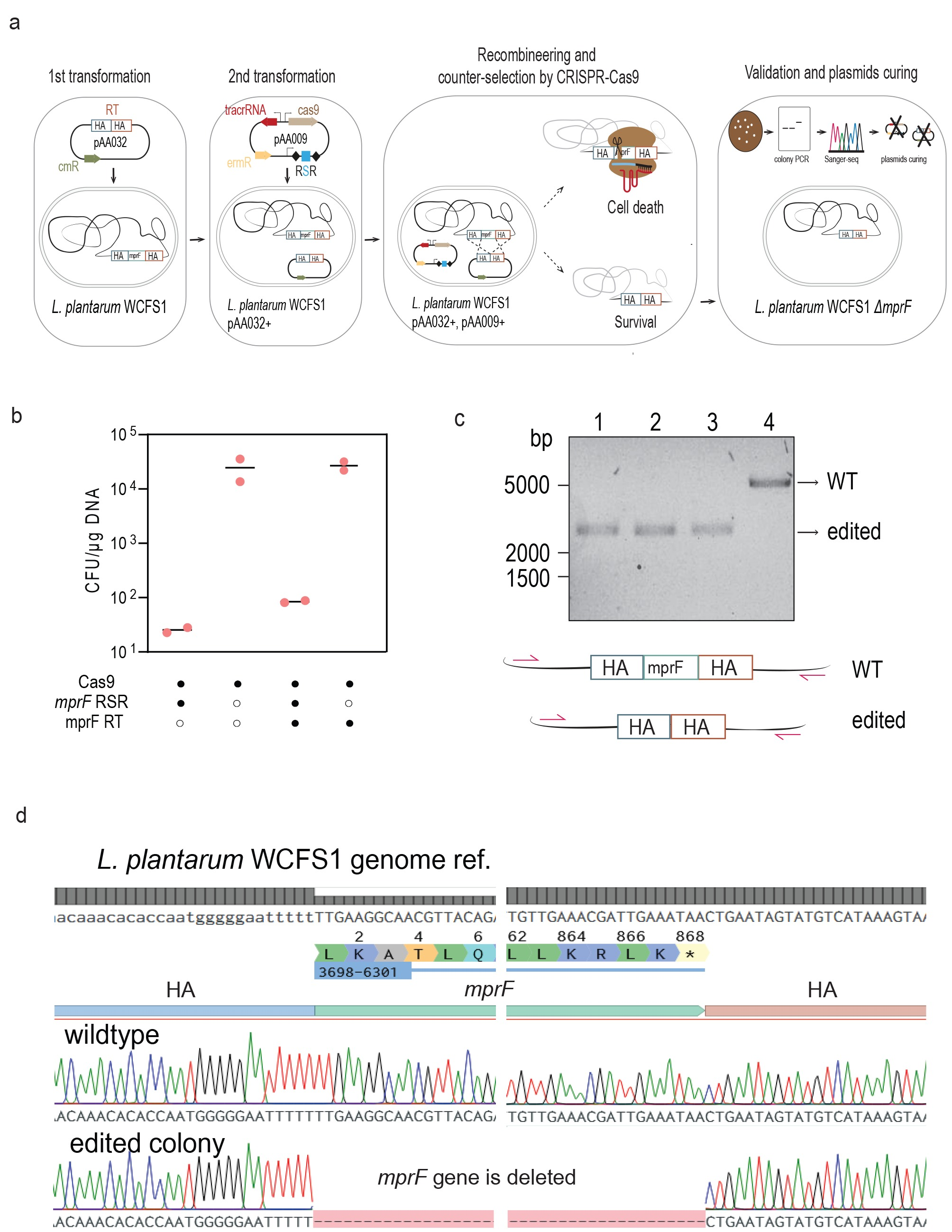

Supplement: S3 Fig — (a) Scheme illustrating CRISPR-Cas9 genome editing approach to knock out mprF in L. plantarum WCFS1. First, a recombineering template (RT) plasmid pAA032 containing approximately 250-bp homology arms (HA9) flanking mprF gene was transformed into L. plantarum. Then, the shuttle vector pAA009 encoding SpyCas9, its tracrRNA, and single-spacer CRISPR array targeting a site within mprF was transformed into the L. plantarum containing recombineering template plasmid to counter-select the unedited cells. Surviving colonies were screened by colony PCR (cPCR) that amplifies the genome of L. plantarum, but not the plasmid with the recombineering template. (b) Killing activity of the guide RNA containing mprF targeting spacer. The observed 1,000-fold reduction in colony forming units (CFU) in the presence of targeting repeat-spacer-repeat (RSR) indicated targeting and cleaving activities on the genome containing mprF gene (c-d) Validation of mprF deletion via colony PCR (c) and Sanger sequencing (d). cPCR was performed using primers that bind to the genome but not the plasmid. PCR products of ~2,400 bp indicated clean deletion of the ~2,600-bp mprF gene. PCR products were then subjected to Sanger sequencing to confirm that mprF gene was successfully deleted from the start codon to the stop codon. After confirmation, plasmids were cured to generate the final strain. (TIF) [file ppat.1012462.s003.tif]

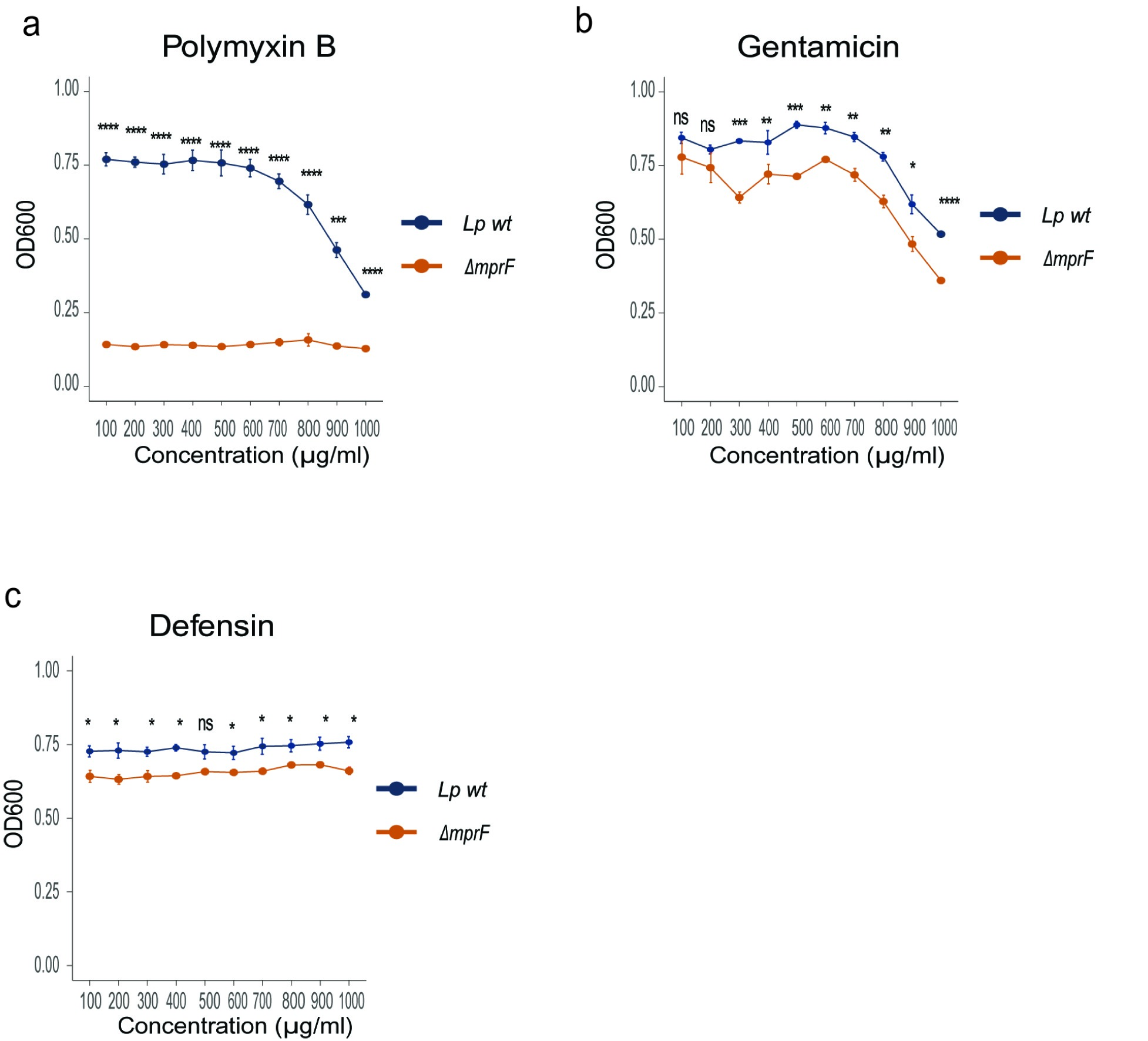

Supplement: S4 Fig — (a-c) Antibiotic Inhibitory Assay (AIA) of L. plantarum wild-type and L. plantarum ΔmprF mutant in MRS media supplemented with Polymyxin B (a), Gentamicin (b), and Defensin (c) (n = 3 independent experiments). Data show mean and SD of 3 independent experiments. (TIF) [file ppat.1012462.s004.tif]

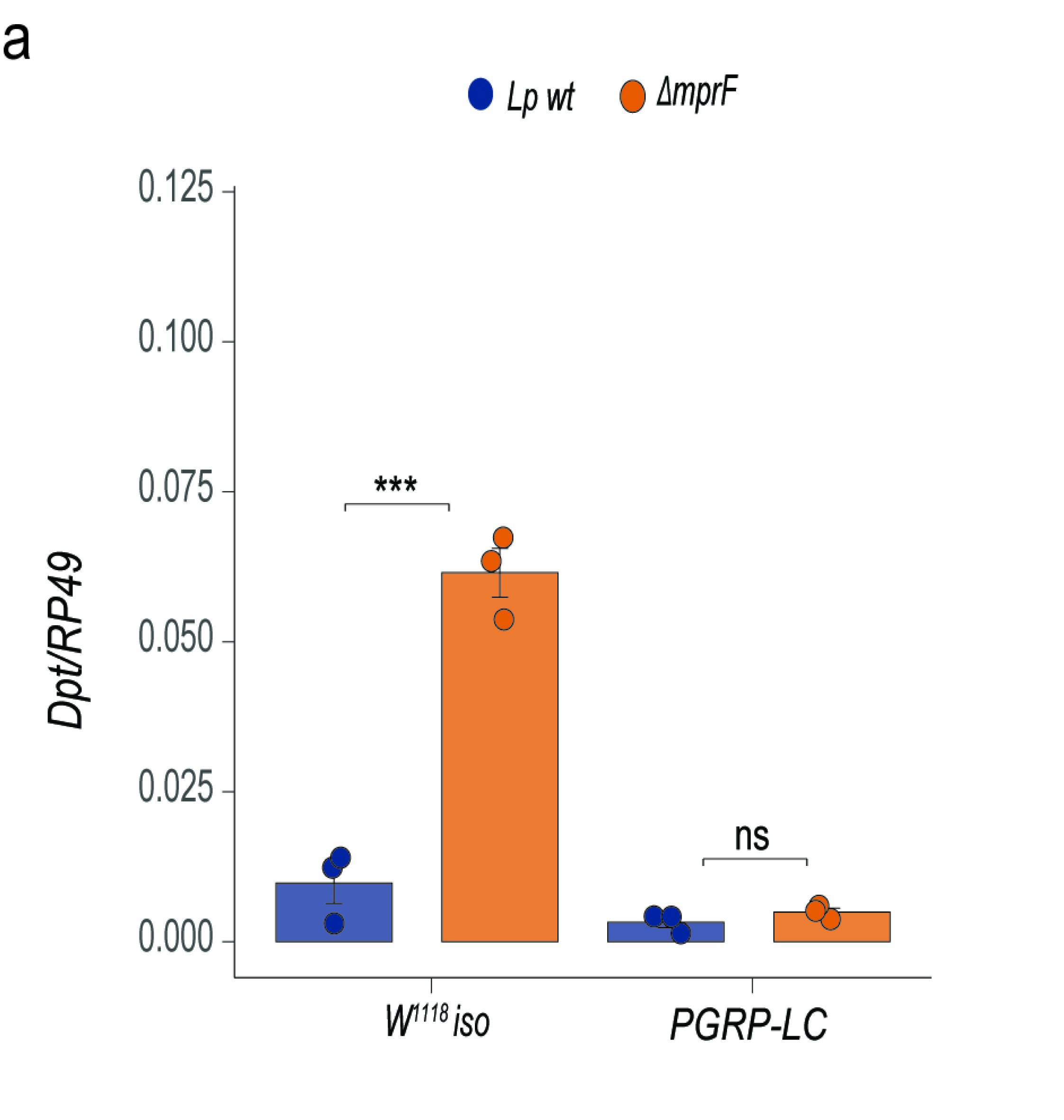

Supplement: S5 Fig — Intestinal Diptericin A gene expression 5 days post colonization with L. plantarum wild-type and ΔmprF mutant in wild-type and PGRP-LC12 flies. Individual dots show gene expression per 20 female guts. Bar plots show mean and SEM. (TIF) [file ppat.1012462.s005.tif]

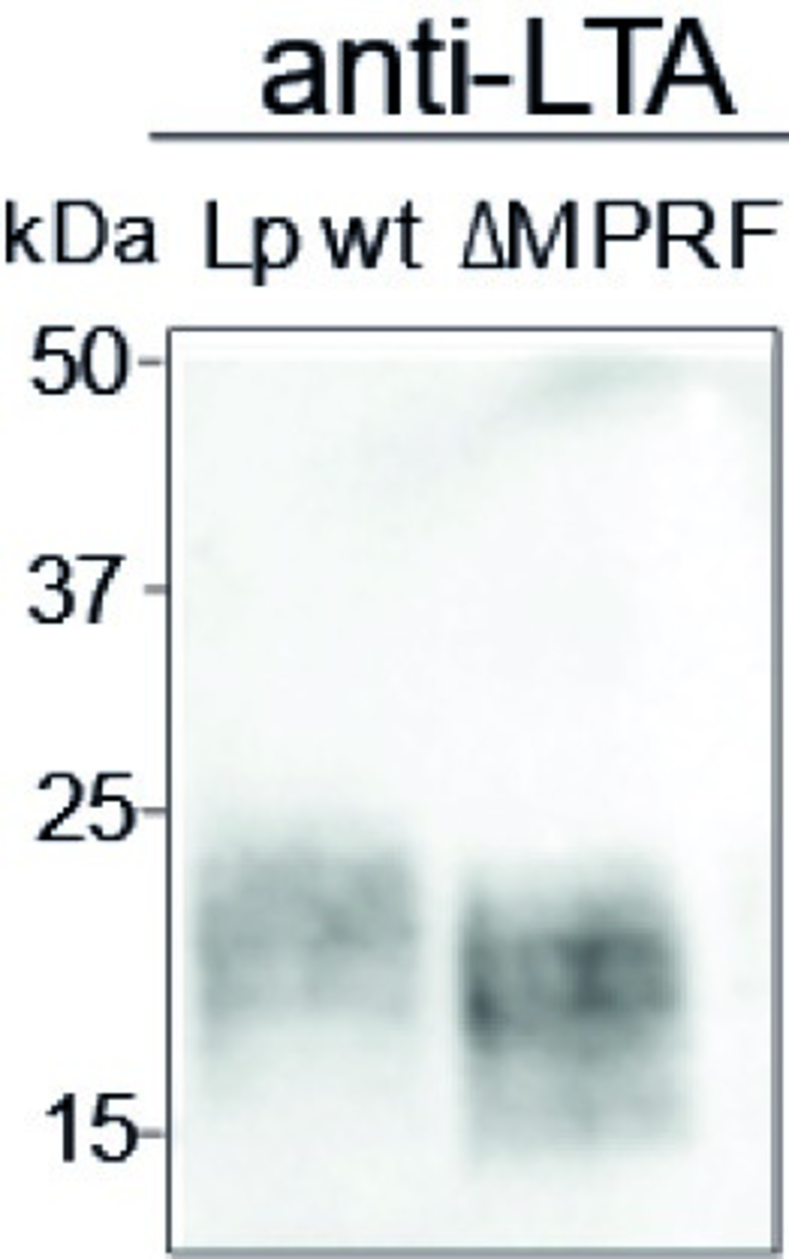

Supplement: S6 Fig — LTA profile of L. plantarum wild-type and ΔmprF mutant detected with Western blot and anti-LTA MAb at a 1:1,000 dilution. Crude cell lysates were used. (TIF) [file ppat.1012462.s006.tif]

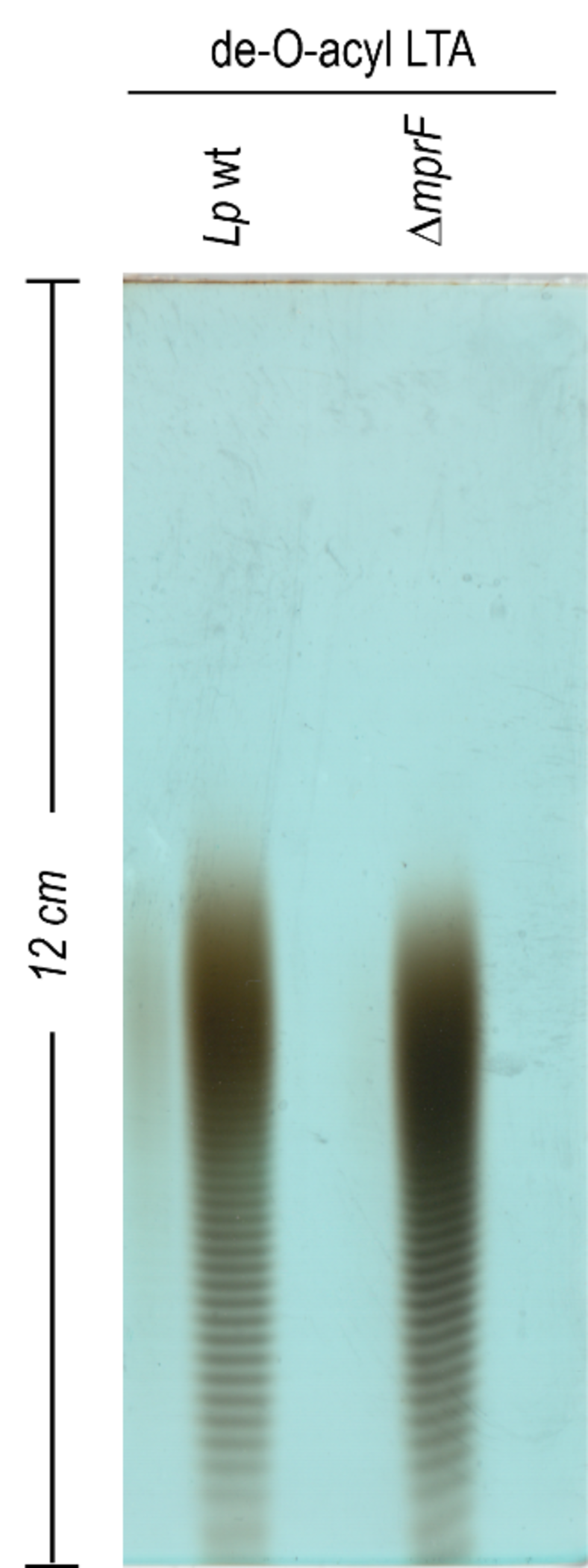

Supplement: S7 Fig — Truncated version of this gel is depicted in Fig 6B. (TIF) [file ppat.1012462.s007.tif]

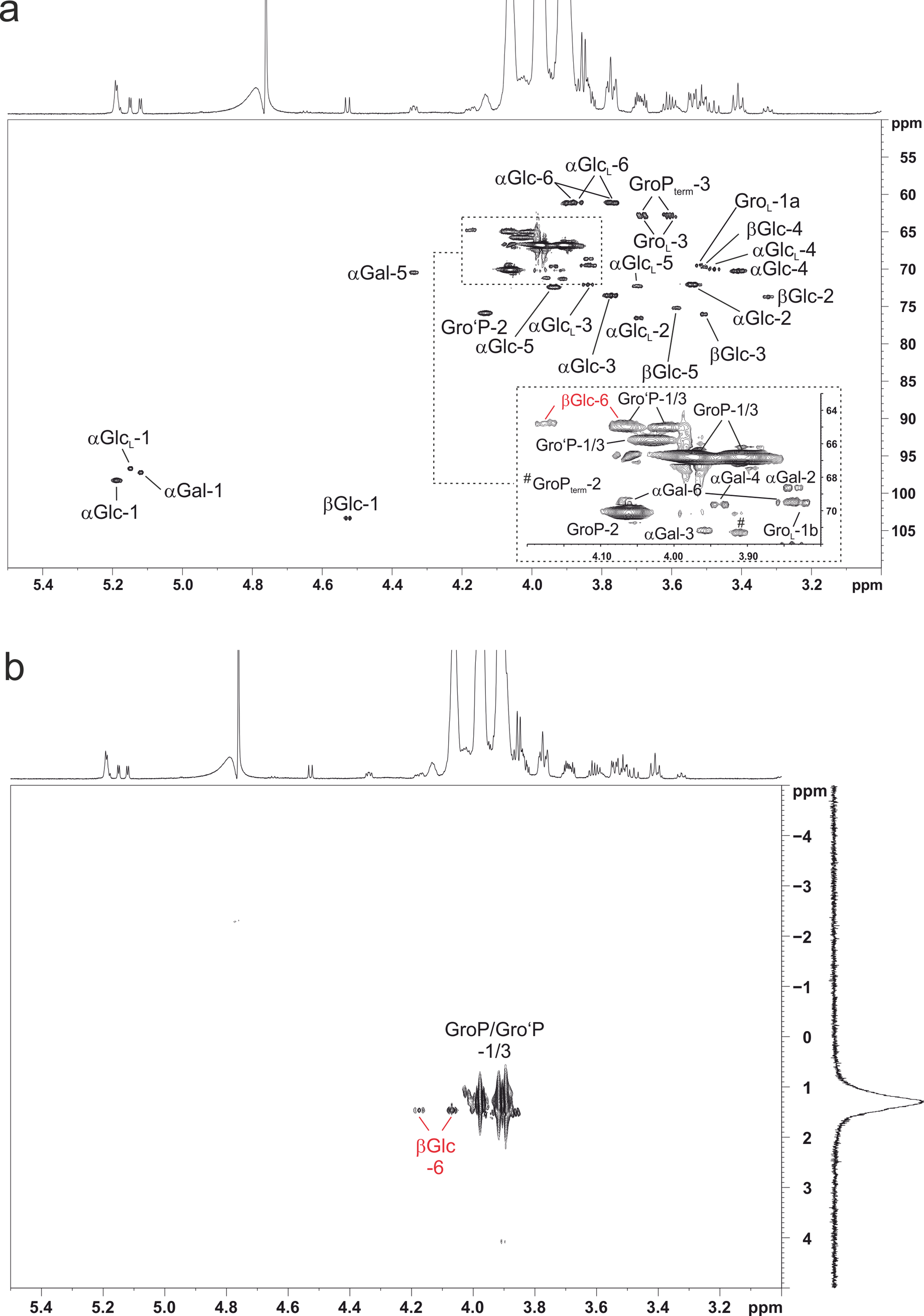

Supplement: S8 Fig — (a) Shown is a section (δH 5.5–3.0 ppm; δC 110–50 ppm) of the 1H,13C-HSQC NMR spectrum (recorded in D2O at 300 K as dept-version) including signal assignment. (b) Shown is a section (δH 5.5–3.0 ppm; δP 5-(–5) ppm) of the 1H,31P-HMQC NMR spectrum (recorded in D2O at 300 K) including signal assignment. In both panels, the cross-correlations for the O-6 position of βGlcp are highlighted in red. The corresponding NMR chemical shift data are listed in S1 Table. (TIF) [file ppat.1012462.s008.tif]

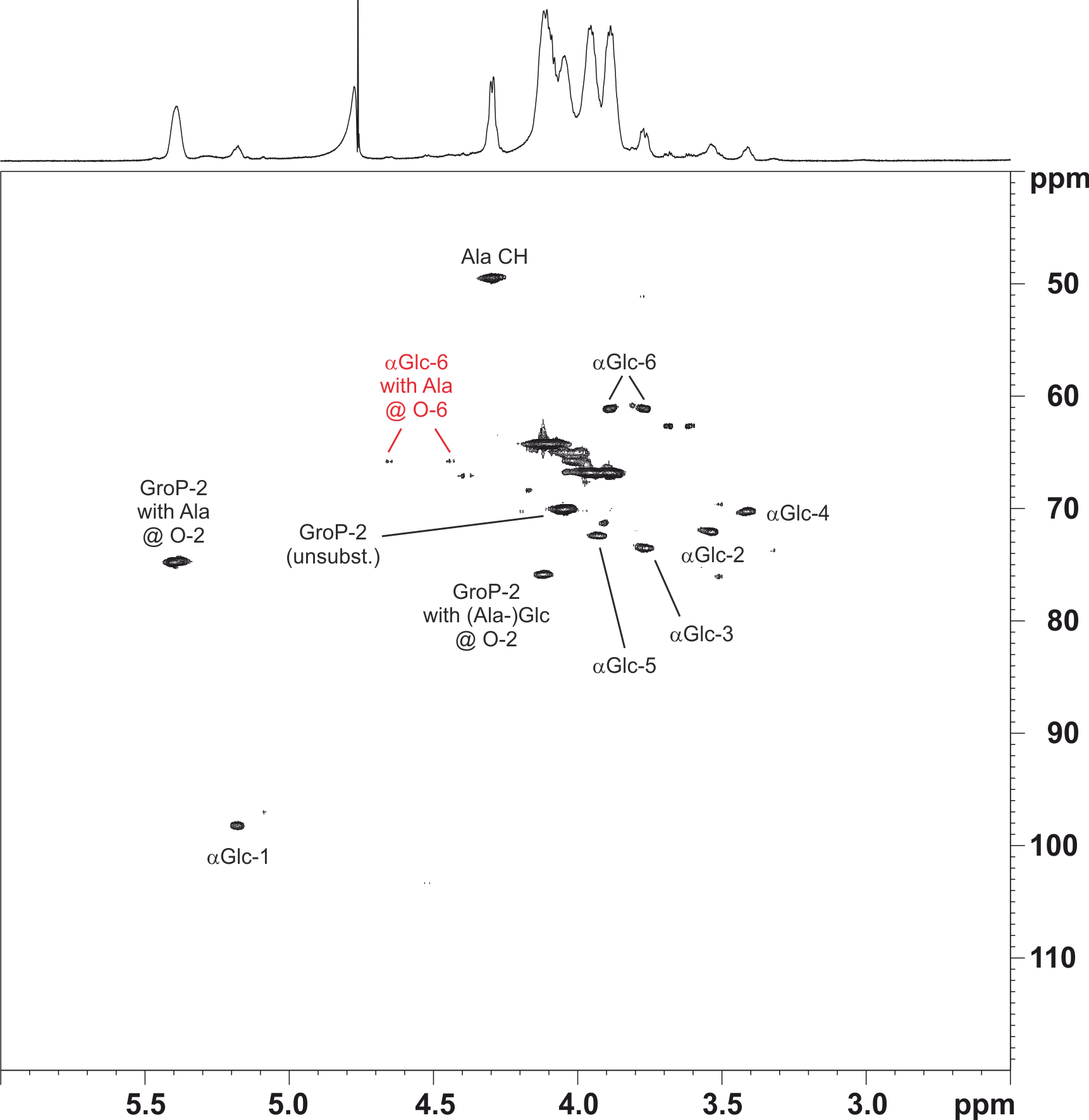

Supplement: S9 Fig — In addition, there is evidence for a small proportion of 6-Ala-αGlcp (as described for Lp strain NC8. Shown is a section (δH 6.0–2.5 ppm; δC 120–40 ppm) of the 1H,13C-HSQC NMR spectrum (recorded in D2O at 300 K as dept-version) obtained from native LTA of the wild-type strain. (TIF) [file ppat.1012462.s009.tif]
