# Supplementary material for: MprF-mediated immune evasion is necessary for Lactiplantibacillus plantarum resilience in the Drosophila gut during inflammation
Source: PLoS Pathog. 2024 Aug 19;20(8):e1012462. doi: 10.1371/journal.ppat.1012462 (PMC11361745; doi:10.1371/journal.ppat.1012462)
Supplement: S1 Table — (DOCX) [file ppat.1012462.s010.docx]

**Table S1.** **^1^H (700.4 MHz), ^13^C NMR (176.1 MHz), and ^31^P NMR (283.5 MHz) chemical shift data (δ, ppm) [*J*, Hz] for *L. plantarum* strain WCFS1 LTA after hydrazine treatment (de-O-acyl LTA) recorded in D_2_O at 300 K.**

| **Residue**  **[assignment]** | **H-1**  ***C-1*** | **H-2**  ***C-2*** | **H-3**  ***C-3*** | **H-4**  ***C-4*** | **H-5**  ***C-5*** | **H-6**  ***C-6*** |
| --- | --- | --- | --- | --- | --- | --- |
| Gro-(1→ [**Gro_L_]** | 3.84-3.81*  3.53.3.50*  69.*6* | *n.d.*  *n.d.* | 3.70-3.67*  3.63-3.59*  *62.8* |  |  |  |
|  |  |  |  |  |  |  |
| →1)-α-Glc*p*-(2→ [α**Glc_L_**] | 5.15 [3.5]  *96.8* | 3.71-3.68*  *76.6* | 3.86-3.82*  *72.2* | 3.50-3.46*  *70.2* | 3.72-3.68*  *72.4* | 3.89-3.85*  3.79-3.75*  *61.2* |
|  |  |  |  |  |  |  |
| →1)-α-Gal*p*-(6→ [α**Gal**] | 5.12 [4.1] | 3.86-3.82* | 3.97-3.94* | 3.95-3.93* | 4.36-4.32* | 4.08-4.04* |
|  |  |  |  |  |  | 3.87-3.82* |
|  | *97.3* | *68.8* | *71.3* | *69.7* | *70.6* | *69.6* |
|  |  |  |  |  |  |  |
| →1)-β-Glc*p*-(6→*P* [βG**lc**] | 4.53 [8.0] | 3.34-3.30* | 3.52-3.49* | 3.52-3.49* | 3.60-3.56* | 4.20-4.16* |
|  |  |  |  |  |  | 4.09-4.05* |
|  | *103.4* | *73.8* | *76.1* | *69.8* | *75.3* | *65.0* |
|  |  |  |  |  |  |  |
| *P*→1)-Gro-(3→ [**GroP**] | 4.01-3.95*  3.94-3.88*  *66.9 [5.2]* | 4.09-4.03*  *70.2* | 4.01-3.95*  3.94-3.88*  *66.9 [5.2]* |  |  |  |
|  |  |  |  |  |  |  |
| *P*→1, αGlc→2)-Gro-(3→  [**Gro’P**]^$^ | 4.06-4.00*  *65.9* | 4.16-4.11*  *76.0* | 4.06-4.00*  *65.9* |  |  |  |
|  |  |  |  |  |  |  |
|  |  | and |  |  |  |  |
|  |  |  |  |  |  |  |
|  | 4.09-4.04* | 4.16-4.11* | 4.09-4.04* |  |  |  |
|  | 4.04-3.99* |  | 4.04-3.99* |  |  |  |
|  | *65.1* | *76.0* | *65.1* |  |  |  |
|  |  |  |  |  |  |  |
| *P*→1)-Gro [**Gro^term^**] | 3.96-3.92*  3.89-3.85*  *67.0* | 3.93-3.90*  *71.4* | 3.70-3.67*  3.63-3.59*  *62.7* |  |  |  |
|  |  |  |  |  |  |  |
| α-Glc*p*-(1→ [α**Glc**] major | 5.19 [3.6] | 3.54 | 3.80-3.75* | 3.41 [9.6, | 3.96-3.92* | 3.92-3.87* |
|  |  | [10.3, 3.6] |  | 9.5] |  | 3.79-3.75* |
|  | *98.3* | *72.2* | *73.6* | *70.3* | *72.5* | *61.2* |
|  |  |  |  |  |  |  |
| minor | 5.18 [3.6] | 3.54-3.51* |  |  |  |  |
|  | *98.4* | *72.2* |  |  |  |  |
|  |  |  |  |  |  |  |
| **^31^P** Gro-*P*-Gro_term_ 1.52^#^; βG**lc**-6-*P*-Gro 1.48^#^; Gro-*P*-Gro / Gro-*P*-Gro‘ / Gro‘-*P*-Gro‘ 1.70-0.95 | | | | | | |

*non-resolved multiplet; ^#^values determined using ^1^H,^31^P-HMQC-TOCSY; ^$^two different signals for Gro’P-1/3 are present due to varying nature of the neighboring repeating units (possible are the combinations 2 x GroP, 1 x GroP + 1 x Gro’P or 2 x Gro’P); *n.d.* = not detected.
