# Supplementary material for: MprF-mediated immune evasion is necessary for Lactiplantibacillus plantarum resilience in the Drosophila gut during inflammation
Source: PLoS Pathog. 2024 Aug 19;20(8):e1012462. doi: 10.1371/journal.ppat.1012462 (PMC11361745; doi:10.1371/journal.ppat.1012462)
Supplement: S2 Table — (DOCX) [file ppat.1012462.s011.docx]

**Table S2.** **Bacterial strains used in this study.**

| **Strain** | **Growth conditions** | **Relevant characteristics** | **Reference or sourse** |
| --- | --- | --- | --- |
| *Pectobacterium carotovorum carotovorum (Ecc15)* | 29°C Shaking incubator, in LB media | Natural *Drosophila* pathogen | Basset et al., 2000 [1] |
|  |  |  |  |
| *Lactiplantibacillus plantarum NCIMB 8826 (WCFS1)* | 37°C Stationary Incubator, in MRS media | Strain with high transformation efficiency | Kleerebeyem et al 2003 [2] |
|  |  |  |  |
| *Lactiplantibacillus plantarum ∆mprF* | 37°C Stationary Incubator, in MRS media | NCIMB strain deleted for mprF gene | This study |
| *L. plantatum ∆mprF pSIP409-Lp-mprF* | 37°C Stationary Incubator, in MRS media supplemented with IP-673 peptide | NCIMB strain deleted for mprF gene, containing pSIP409::mprF plasmid for MprF overexpression | This study |
| *Staphylococcus aureus 113* | 37°C Shaking incubator, in TSB media | Pathogen use for Drosophila systemic infection | Peschel et al., 2001 [3] |
| *Staphylococcus aureus ∆mprF* | 37°C Shaking incubator, in TSB media | S.aureus 113 strain deleted for MPRF genes | Peschel et al., 2001 [3] |
|  |  |  |  |
|  |  |  |  |
| *E. coli TOP10* | 37°C Shaking incubator, in LB media | F–mcrA Δ(mrr-hsdRMS-mcrBC) φ80lacZΔM15 ΔlacX74 recA1 araD139 Δ(ara-leu)7697 galU galK λ–rpsL(StrR) endA1 nupG | Thermo Fisher Scientific |
|  |  |  |  |
|  |  |  |  |
|  |  |  |  |
| *E. coli-pBAD18-LpMprF* | 37°C Shaking incubator, in LB media suplemented with Ampicillin and L-arabinose 0.2% | E. coli TOP10 strain, containing pBAD18 plasmid for LpMprF overexpression | This study |
|  |  |  |  |
|  |  |  |  |
| *E. coli DH5a* | 37°C Shaking incubator, in LB media | *F– φ80lacZΔ M15 Δ (lacZYA-argF) U169 recA1 endA1 hsdR17 (rK– mK+) phoA supE44 λ- thi–1 gyrA96 relA1* | Thermo Fisher Scientific |
|  |  |  |  |
|  |  |  |  |
|  |  |  |  |
| *E. coli EC135* | 37°C Shaking incubator, in LB media | *recA1 recA+ mcrA Δ(mrr-hsdRMS-mcrBC) Δdcm::FRT Δdam::FRT* | Zhang et al., 2012 [4] |
|  |  |  |  |
|  |  |  |  |

**References**

1. Basset a, Khush RS, Braun a, Gardan L, Boccard F, Hoffmann J a, et al. The phytopathogenic bacteria Erwinia carotovora infects Drosophila and activates an immune response. Proc Natl Acad Sci U S A. 2000;97: 3376–81. doi:10.1073/pnas.070357597

2. Kleerebezem M, Boekhorst J, Van Kranenburg R, Molenaar D, Kuipers OP, Leer R, et al. Complete genome sequence of Lactobacillus plantarum WCFS1. Proc Natl Acad Sci U S A. 2003;100: 1990–1995. doi:10.1073/PNAS.0337704100

3. Peschel A, Jack RW, Otto M, Collins LV, Staubitz P, Nicholson G, et al. Staphylococcus aureus Resistance to Human Defensins and Evasion of Neutrophil Killing via the Novel Virulence Factor Mprf Is Based on Modification of Membrane Lipids with l-Lysine. J Exp Med. 2001;193: 1067–1076. doi:10.1084/JEM.193.9.1067

4. Zhang G, Wang W, Deng A, Sun Z, Zhang Y, Liang Y, et al. A Mimicking-of-DNA-Methylation-Patterns Pipeline for Overcoming the Restriction Barrier of Bacteria. PLOS Genet. 2012;8: e1002987. doi:10.1371/JOURNAL.PGEN.1002987
