# Supplementary material for: MprF-mediated immune evasion is necessary for Lactiplantibacillus plantarum resilience in the Drosophila gut during inflammation
Source: PLoS Pathog. 2024 Aug 19;20(8):e1012462. doi: 10.1371/journal.ppat.1012462 (PMC11361745; doi:10.1371/journal.ppat.1012462)
Supplement: S3 Table — (DOCX) [file ppat.1012462.s012.docx]

**Table S3. Plasmids used in this study.**

| **CBS ID** | **Plasmid ID** | **Description** | **Plasmid map** | **Source** | **Use** | **Resistance** |
| --- | --- | --- | --- | --- | --- | --- |
|  |  |  |  |  |  |  |
| CBS-4146 | pCB591 | Backbone for RT plasmid | <https://benchling.com/s/seq-EZ0vHyRUqOoWOlH3zF4P?m=slm-ll8mCsgv7yZSqDWPZHxF> | Leenay et al., 2019 [1] | Cloning | Amp100/Cm10 |
| CBS-4100 | pCB578 | Cas9 + tracrRNA + *ackA* RSR backbone for targeting plasmid | <https://benchling.com/s/seq-CE1TOCpvMS7aVMML8oUC?m=slm-e6Ha9sWlj5BClVFEERtN> | Leenay et al., 2019 [1] | Cloning | Erm300/Erm10 |
| CBS-4101 | pCB577 | Cas9 + tracrRNA without RSR | <https://benchling.com/s/seq-9KP7wflTR5qRk42gaxba?m=slm-Pz6BgawZrpk50JCTtDZZ> | Leenay et al., 2019 [1] | Fig.S3 | Erm300/Erm10 |
| CBS-4148 | pAA032 | Final RT for clean deletion *mprF* in *L.plantarum* WCFS1_250-bp upstream HA + 250-bp downstream HA | <https://benchling.com/s/seq-ahIHCDXtqhxqzi6OdnRW?m=slm-hlx6hw9Mzes5ovINbI1W> | This study | Fig.S3 | Amp100/Cm10 |
| CBS-3446 | pAA009 | SpyCas9 + tracrRNA + RSR for *mprF L.plantarum* WCFS1 | <https://benchling.com/s/seq-m6uWlM3tLRhdvmf6GbEg?m=slm-QCQffVNSbwwVnAgIjiSM> | This study | Fig.S3 | Erm300/Erm10 |

**References**

1. Leenay RT, Vento JM, Shah M, Martino ME, Leulier F, Beisel CL. Genome Editing with CRISPR-Cas9 in Lactobacillus plantarum Revealed That Editing Outcomes Can Vary Across Strains and Between Methods. Biotechnol J. 2019;14: 1700583. doi:10.1002/BIOT.201700583
