# Supplementary material for: MprF-mediated immune evasion is necessary for Lactiplantibacillus plantarum resilience in the Drosophila gut during inflammation
Source: PLoS Pathog. 2024 Aug 19;20(8):e1012462. doi: 10.1371/journal.ppat.1012462 (PMC11361745; doi:10.1371/journal.ppat.1012462)
Supplement: S4 Table — (DOCX) [file ppat.1012462.s013.docx]

**Table S4. Primers used in this study.**

| **Primer name** | **Reference or description** | **Sequence (5‘ – 3‘)** |
| --- | --- | --- |
| Defensin F | This study | AGTTCTTCGTTCTCGTGGCT |
| Defensin R | This study | CCACATCGGAAACTGGCTGA |
| Drosomycin F | Dudzic et al 2019 [1] | CGTGAG AACCTTTTCCAATATGAT |
| Drosomycin R | Dudzic et al 2019 [1] | TCCCAGGACCACCAGCAT |
| RP49 F | Iatsenko et al 2018 [2] | GACGCTTCAAGGGACAGTATCTG |
| RP49 R | Iatsenko et al 2018 [2] | AAACGCGGTTCTGCATGAG |
| DptA F | Iatsenko et al 2018 [2] | TGGTGGAGTGGGCTTCAT |
| DptA R | Iatsenko et al 2018 [2] | GCTGCGCAATCGCTTCTA |
| PGRP-LB F | This study | GGCATGATTTACACCGGCAG |
| PGRP-LB R | This study | TCTCCGATCAGCACAATGCC |
| Pirk F | This study | GCTGCAATGGACTGCTCAAG |
| Pirk R | This study | AGAGCTGGGCCTTTTCTTGG |
| oAA027 | Oligo for inserting *mprF* target spacer FWD | CGACGGAAGTCGGCAAAATTGCGAGTTTTAGAGCTATGCTGTTTTGAATGGTCCCAAAACATGC |
| oAA028 | Oligo for inserting *mprF* target spacer REV | GGCCGCATGTTTTGGGACCATTCAAAACAGCATAGCTCTAAAACTCGCAATTTTGCCGACTTCCGTCGAT |
| oAA097 | Primer for amplifying the genome region of WCFS1 containing *mprF* gene and 250-bp upstream and downstream FWD | ACATAAACGGTAAAGGTTGGTAAAGC |
| oAA098 | Primer for amplifying the genome region of WCFS1 containing *mprF* gene and 250-bp upstream and downstream REV | AGGCTAACCTCGACCTATTC |
| oAA094 | Primer for amplifying the backbone for RT plasmid FWD | GAATAGGTCGAGGTTAGCCTGCTCAAGCTTTCTTTGAACC |
| Pirk R | This study | AGAGCTGGGCCTTTTCTTGG |
| oAA099 | Primer for amplifying the backbone for RT plasmid REV | CCAACCTTTACCGTTTATGTAACTAGTGGTACCTTAGCTGTCAGC |
| oAA033 | Primer to remove *mprF* from recombineering template plasmid FWD | CTGAATAGTATGTCATAAAGTAAG |
| oAA034 | Primer to remove *mprF* from recombineering template plasmid REV | AAAAATTCCCCCATTGGTG |
| oAA038 | Primer on the plasmid backbone for amplifying the RT region for colony PCR FWD | TTTTGCTCACATGTTCTTTC |
| oAA039  oAA036 | Primer on the plasmid backbone for amplifying the RT region for colony PCR REV  Primer on genome for cPCR of mutant FWD | CTGCTTTTTGGCTATCAATC  CGTCAGTTGCTTGTCATTAT |
|  |  |  |
| oAA037 | Primer on genome for cPCR of mutant REV | AAAGACCGTTCTGAAAAGCA |
| oAA047 | Primer on genome for sequencing FWD | CGAGTTTTTTTGCATGGTGCATAAG |
| oAA130 | Primer on genome for sequencing REV | ACGACGATCTAGTCGCCATG |
| mprF F BamHI | Forward primer for *mprF* cloning into pBAD18 plasmid | CGCGGATCCGATGAAGGCAACGTTACAGAAA |
| mprF R SalI | Reverse primer for *mprF* cloning into pBAD18 plasmid | ACGCGTCGACTTATTTCAATCGTTTCAACAACCAT |
| mprF NcoI F | Forward primer for *mprF* cloning into pSIP409 plasmid | TAAGCACCATGGatgaaggcaacgttacagaa |
| mprF XhoI R | Reverse primer for *mprF* cloning into pSIP409 plasmid | TAAGCACTCGAGttatttcaatcgtttcaaca |

**References**

1. Dudzic JP, Hanson MA, Iatsenko I, Kondo S, Lemaitre B. More Than Black or White: Melanization and Toll Share Regulatory Serine Proteases in Drosophila. Cell Rep. 2019;27: 1050-1061.e3. doi:10.1016/j.celrep.2019.03.101

2. Iatsenko I, Boquete J-P, Lemaitre B. Microbiota-Derived Lactate Activates Production of Reactive Oxygen Species by the Intestinal NADPH Oxidase Nox and Shortens Drosophila Lifespan. Immunity. 2018;49: 929-942.e5. doi:10.1016/J.IMMUNI.2018.09.017
